# Clinician identified barriers to treatment for individuals in Appalachia with opioid use disorder following release from prison: a social ecological approach

**DOI:** 10.1186/s13722-018-0124-2

**Published:** 2018-12-03

**Authors:** Amanda M. Bunting, Carrie B. Oser, Michele Staton, Katherine S. Eddens, Hannah Knudsen

**Affiliations:** 1 Department of Sociology, University of Kentucky, Lexington, USA; 2 Center on Drug and Alcohol Research, University of Kentucky, Lexington, USA; 3 Department of Behavioral Science, University of Kentucky, Lexington, USA; 4 Indiana University Network Science Institute, Indiana University, Bloomington, USA

**Keywords:** Opioid use disorder, Barriers to treatment, Rural, Appalachia, Reentry, Treatment, Qualitative research

## Abstract

**Background:**

The non-medical use of opioids has reached epidemic levels nationwide, and rural areas have been particularly affected by increasing rates of overdose mortality as well as increases in the prison population. Individuals with opioid use disorder (OUD) are at increased risk for relapse and overdose upon reentry to the community due to decreased tolerance during incarceration. It is crucial to identify barriers to substance use disorder treatment post-release from prison because treatment can be particularly difficult to access in resource-limited rural Appalachia.

**Methods:**

A social ecological framework was utilized to examine barriers to community-based substance use treatment among individuals with OUD in Appalachian Kentucky following release from prison. Semi-structured qualitative interviews with 15 social service clinicians (SSCs) employed by the Department of Corrections were conducted to identify barriers at the individual, interpersonal, organizational/institutional level, community, and systems levels. Two independent coders conducted line-by-line coding to identify key themes.

**Results:**

Treatment barriers were identified across the social ecological spectrum. At the individual-level, SSCs highlighted high-risk drug use and a lack of motivation. At the interpersonal level, homogenous social networks (i.e., homophilious drug-using networks) and networks with limited treatment knowledge inhibited treatment. SSC’s high case load and probation/parole officer’s limited understanding of treatment were organizational/institutional barriers. Easy access to opioids, few treatment resources, and a lack of community support for treatment were barriers at the community level. SSC’s noted system-level barriers such as lack of transportation options, cost, and uncertainty about the implementation of the Affordable Care Act.

**Conclusions:**

More rural infrastructure resources as well as additional education for family networks, corrections staff, and the community at large in Appalachia are needed to address barriers to OUD treatment. Future research should examine barriers from the perspective of other key stakeholders (e.g., clients, families of clients) and test interventions to increase access to OUD treatment.

## Background

The prevalence of individuals with an opioid use disorder (OUD) has increased over the past decade [1–3]. The opioid epidemic has resulted in other negative consequences, including increasing rates of overdose mortalities, emergency room visits, and HIV/HCV prevalence [4–6]. It is estimated that from 2001 to 2016, the additional spending on health care, social services, and the criminal justice system coupled with the associated costs of loss of lives from overdose have reached 1 trillion dollars [7]. More than half of individuals with OUD have contact with the criminal justice system [8], highlighting the crucial need to understand treatment access among this vulnerable population.

While the entire nation is faced with a public health epidemic, certain geographic areas have experienced disproportionate levels of OUD, overdose fatalities, and HIV/HCV seropositive status [5, 9]. At the epicenter of the opioid epidemic is rural Appalachia. The Appalachian states of Kentucky, Ohio, and West Virginia rank among the top five states for age-adjusted opioid overdose deaths [10]. The region also suffers disproportionate rates of opioid related HIV and HCV transmission [5, 11] as use of opioids exceeds national averages [9, 11, 12]. Despite the heavy use of opioids, the economic deprivation specific to Appalachia (i.e., low formal education, high rates of unemployment related to the economy) [11, 13] creates significant challenges to accessing appropriate care. While medications for addiction treatment (MAT) are an effective treatment for OUD, Appalachian communities face barriers to access similar to other rural areas such as lack of prescribing physicians and long waiting lists [14–16]. The rurality of Appalachia creates challenges in accessing substance use disorder treatment due to inadequate transportation [11, 14, 17–19] and a general lack of health-related services [19, 20]. These barriers can be especially pronounced among re-entering populations who already face community reintegration challenges [21, 22].

Disproportionate rates of opioid use exist among criminal justice-involved individuals [23, 24]. Coupled with complex substance histories, opioid misuse is associated with increased involvement in the criminal justice system. OUD is associated with criminal justice involvement due to illicit drug use and the co-occurring criminal activity, as well as through economically motivated crimes to obtain opioids [25, 26]. Providing treatment for OUD among criminal justice-involved individuals is critical, given associated reductions in drug use, crime, and associated costs [27, 28]. The need for treatment is ever more pressing as individuals returning home from prisons face an 129 times greater risk of drug overdose death compared to the general public [29]. However, individuals typically return home to limited services; a situation exacerbated by limited post-release resources [30, 31]. Ensuring recently released populations with substance use disorders are matched with appropriate care is a crucial public health concern.

The current study sought to understand the barriers to treatment for OUD among individuals reentering communities in Appalachia from clinicians’ perspectives. While some research has examined perceived barriers to MAT from the perspectives of clinicians and criminal justice agencies [32, 33], limited research has considered counselor or clinician perspectives to barriers to care in rural areas [19, 34, 35], with no known studies considering the additional vulnerability of criminal justice status. To assist in understanding these barriers, the current study was guided by the social ecological framework [36, 37]. The social ecological model of health proposes that behavior (i.e., the outcome) is determined by five nested levels: intrapersonal factors (i.e., characteristics of the individual), interpersonal factors (i.e., social networks), institutional factors (i.e., social institutions and organizations with formal and informal rules), community factors (i.e., relationships among organizations and institutions), and public policy (i.e., local, state, and national law and policies). The social ecological model suggests behavior is influenced by the social environment [37]. Assessing behavioral outcomes as affected by the various levels improves opportunities for interventions attuned to the appropriate level-specific factors. Utilization of all five levels of the framework provides the additional advantage of allowing researchers to assess the interaction of various levels and provide more comprehensive suggestions. Many health interventions focus on the individual or interpersonal level, and multilevel interventions or interventions at upper ecological levels are critically lacking [38]. The current research used the five levels of the social ecological model to identify barriers to OUD treatment through qualitative interviews with social service clinicians who work with Appalachian individuals re-entering society after incarceration. These clinicians are uniquely qualified to describe such barriers because they routinely work with re-entering individuals and provide post-release linkages to care.

## Methods

### Data collection

All social service clinicians (SSCs) employed in Appalachian counties in Kentucky (n = 15) were invited to participate in the study. The 15 SSCs covered a service area of 54 Appalachian counties [39]. SSCs are employed by the Kentucky Department of Corrections (DOC) to provide assessments and referrals to treatment following release for substance involved parolees. Typically, SSCs meet with re-entering individuals within 72 h of their release. All SSCs who were invited to participate provided informed consent and were interviewed. Four of the 15 SSCs had additional supervisor duties, which includes administrative responsibilities and clinical supervision.

### Design

Recorded qualitative interviews by a trained interviewer took place between January and April of 2017 during regular office hours, and in the office of the SSC. All interviews were conducted by the same interviewer, who has a Master’s degree in Public Administration and over a decade of experience conducting qualitative interviews with DOC staff and administrators on other federally funded projects. Interviews were guided by the social ecological framework to explore challenges to OUD treatment in Appalachia related to client characteristics, social networks, the DOC, substance use disorder treatment, and the health care system. SSC’s views on MAT were also assessed. Informed consent was obtained and interviews averaged 39 min (range: 26–64 min). Participants were not allowed monetary compensation as part of DOC guidelines, thus a picture frame valuing less than $20 was provided as a token of appreciation. The study was approved by a university’s Institutional Review Board and participants were protected by a federal Certificate of Confidentiality.

### Data analysis

The interviews were transcribed verbatim. Identifying information (i.e., names of individuals, cities, counties) was removed. Two researchers conducted line-by-line coding of each interview independently to generate initial codes. Consensus was then used to reach agreement on the primary codes. Disagreement about codes or themes were resolved by discussion among the two coders and re-review of the original transcripts. Following thematic analysis techniques [40], the codes were then organized thematically according to the social ecological framework.

## Results

All respondents were white females, the majority of whom had a Master’s degree (73.33%, n = 11). SSCs had been employed in their current position for an average of 5.5 years, with lengths of tenure ranging from 2 months to 22 years. One-third (33.3%, n = 5) of the SSCs had previously been employed in corrections as a parole/probation or a correctional officer, and a little over one-fourth (26.7%, n = 4) had previously worked as a clinician in a substance use disorder treatment center.

### Individual level barriers

*High risk use* Several SSCs (n = 5) discussed OUD clients as being significantly more challenging than clients of the past or clients without OUD. Injection drug use and the increased risk for overdose made this current generation of clients particularly high-risk in the eyes of the SSCs. The clinicians also referred to the high rate of relapse associated with OUD. Conceptualization of their clients as being at high risk of negative outcomes was additionally tied to the young age and limited life experience of clients, as expressed by one SSC supervisor:

And a lot of times they’re high risk substance abuser because they tend to use, IV [intravenous] drug users a lot of time. And over the course of my career it seems like 10 years ago, 15 years ago you would not have an IV user until they were in their late 20 s, early 30 s. Like we didn’t see it. And now they’re coming into the system at 18 and 19 as an IV drug user.

SSC Supervisor #3

*Stigma and lack of motivation* Over half (n = 8) of the SSCs referred to specific client characteristics as preventing OUD treatment engagement and subsequent recovery. For these SSCs, clients were stated to be dishonest and/or lack the motivation necessary to succeed in recovery. Some SSCs stated their clients were unwilling to participate, or frequently would use other barriers as excuses to not participate in services such as SSC aftercare programs or MAT. Sometimes lack of honesty was thought to stem from embarrassment and stigma, especially among individuals whose OUD developed from a legitimate medical need:

I think a lot of times they’re embarrassed and don’t want to be honest. There are a lot of clients that started out that they were prescribed medications, and I think they’re very shocked to find themselves in this situation and that’s an embarrassment and they don’t want to be honest about that.

SSC # 15

### Interpersonal level barriers

*Homophilious social networks* Most SSCs noted that drug use was considered normative among the friends and families of clients (n = 12) indicating homophilious social networks. Homophily refers to the homogeneity of characteristics among individual’s personal networks [41]. Further, networks tended to remain the same pre- and post-reentry. This stability of networks, combined with normative drug use, was discussed at length by SSCs. The homophilious networks threatened clients’ likelihood of recovery, and were observable within the DOC and community at large, as explained here:

It seems that everyone, everyone they know or associate with has been or is currently facing the challenge of their addiction, the challenges of their addictions as well. They don’t have sober friends or a sober support system. And a lot of it is generational use. Even their family are not a good support system because it’s been so embedded.

SSC Supervisor #4

*Networks have limited knowledge of treatment* In addition to social networks being homophilious, SSCs stated that client networks had misperceptions about MAT (n = 7), particularly when family members had negative perceptions of pharmacotherapy, as illustrated by the following SSC statement:

The families are like, ‘it’s just another drug that you’re on, I don’t know why you’re on it, you don’t need to be on anything else, you’ve been clean for years or months’- however long they stayed in the jail or the institution, ‘you don’t need to be on that.’

SSC #14

The normativity of drug use in the clients’ networks (i.e., homophilious networks), created a lack of knowledge on how to best promote recovery for clients’ OUD. Specifically, it was perceived that the networks and clients themselves, do not prioritize certain places or behaviors that could best promote recovery among clients. As explained by one SSC supervisor:

So it’s like, well if they’re not using heroin or if they’re not using opioids then alcohol’s not going to hurt them because that’s not what got them in trouble, or that’s not what they OD’d [overdosed] on. And so then what’s the, social norms sometimes get skewed by their family. And the farther removed you are from having a bigger, I guess network of people to associate with, your abnormal looks normal.

SSC Supervisor #3

Thus, the lack of knowledge and perceptions of people with whom clients have social relationships after their release from prison present challenges not only to accessing treatment, but also to succeeding even when treatment was made available.

### Institutional and organizational level barriers

*High SSC caseload* A majority (n = 9) of SSCs stated that large caseloads made their job difficult and was a significant barrier in providing services to their clients. High caseloads, coupled with responsibility for large geographic areas spread across multiple counties, resulted in limited time with each client with months between appointments. Consider the struggles as stated by one SSC:

However just time to get to your mobile sites is a barrier. It takes a lot of time, you’ve got to get up early. Even two to three hours earlier. Go there. And of course your schedule is full for even next month, two months, even three months. So you might have someone test positive for heroin or even, let’s say, oxycodone- but you don’t see them for two months from now.

SSC #14

For this particular SSC, the use of e-mail allowed her to close some of the time gaps in her previously two-month long waiting list. The SSC utilized e-mail for initial assessments to determine if clients needed service recommendations for clients with active drug use or if clients were simply complying with DOC regulations and/or judge mandates to receive an assessment. Other SSCs mentioned being accessible by phone or relying on the probation and parole officers to assess and screen those with the most immediate needs for referral. One SSC explained her relationship with the parole and probation officers in the following statement:

So I do my best to try to make sure that they [the officers] know kind of triage, so they know who needs to get on the phone right away, who can wait a week and go on my schedule when I make it back to that county.

SSC # 10

*Lack of parole/probation officer substance use disorder education* While some SSCs relied on probation and parole officers, many discussed the officers lacked a general understanding of OUD, treatment options, and particularly misunderstood MAT. Ten of the SSCs mentioned that probation and parole officers needed more education on substance use disorders in general and/or MAT. Additionally, four of the SSCs stated probation and parole officers would not allow their clients to receive MAT. Three of these referred to buprenorphine specifically, and one discussed prohibition of methadone. One SSC explained how she saw the negative perceptions of MAT among officers:

Unfortunately, I feel like a lot of the treatment around here is just going and getting the medication and unfortunately I think that’s where the judges got the bad taste in their mouths for Suboxone^®^ which bled over to our officers, because they saw so much abuse and so little success stories. But the way it works usually, and what I try to tell the officers is, when you have success stories they aren’t walking in our offices with new charges because they’re a success story so all we see if those clients who are not using successfully.

SSC #10

Despite often sharing an office with the probation and parole officers, SSCs received completely different training, and to their knowledge, the officers did not receive any training on MAT or substance use disorders. There appeared to be a bureaucratic disconnect in who should be providing education for the officers, as some SSCs mentioned educating officers in their offices while others mentioned the need for officers to receive trainings. SSCs appeared willing to help officers when presented with questions as illustrated by the following SSC when asked about her suggestions for improvements:

Education for the clinicians and even the parole officers because they play a role in it too. I think all of us need to work together and so that we can work together for the benefit of the client. Because a lot of the officers, don’t even know what it is. They was asking me about it earlier this morning-what is Vivitrol^®^- and I explained it to them, what it is. But I think that if they, if their department kind of was educated about it, and present to them, it would help them more too.

SSC #8

Given the high caseload and many counties of the SSCs, it is not unsurprising that a disconnect existed in who should be providing education for the officers.

### Community level barriers

Three themes related to community level barriers emerged in the interviews. Barriers were classified as community-level if they were considered to exist as a direct result of the Appalachian communities in which the clients reside.

*Easy access to opioids* Three clinicians discussed the ease of drug availability as a significant barrier to engaging clients in OUD treatment. While not all SSCs referred directly to ease of access to opioids as a barrier, all but one stated that opioid use was rampant among their client caseloads. Some referred to clients who may have started with medically necessary prescription opioids and then transitioned to illicit use. One SSC relayed the following details, highlighting how economic strain among her clients led them to use heroin:

For instance if somebody’s spending three hundred eighty dollars a day for four OxyContins and you can run up to [large urban city] and get a packet of heroin for eighty-five dollars, you know what are you going to do. And that lasts a day and a half, what are you going to do.

SSC #11

Transitioning from non-medical prescription opioid use to heroin is often driven by economic factors and availability but has significant public health impacts, because the purity of heroin is often unknown which can lead to increased risk for overdose.

*Limited availability of treatment resources* Nearly all of the SSCs expressed frustration over the limited availability of treatment resources in Appalachia (n = 13). Limited resources referred to the lack of physicians providing treatment, the limited amount of specialty inpatient and outpatient treatment programs, long waiting lists, and the limited availability of self-help group meetings. Often provision of treatment in the community was only available through DOC involvement with the support of an SSC, as explained by an SSC supervisor:

But if you go to the more rural areas in the Appalachia, it may take you a 20 mile drive to get to your closest AA [Alcoholics Anonymous] meeting, and that’s at a minimum. If you need a [comorbid care center], you have one or two options. Inpatient, well it’s not impossible if they’re going through us. If they’re going through the Department of Corrections we can get them treatment in a relatively decent time. But if they’re just a regular Joe on the streets, it’s really hard.

SSC #1

The SSCs expressed frustration at the lack of resources that they faced in rural areas, which were particularly acute for individuals with co-occurring mental health needs:

This weekend I faced a crisis with a client that was terminated from the recovery center due to suicidal ideations, and nobody was at work, and the hospital didn’t want to take them. The only crisis center would only keep them for 23 h. So here I was with a client who has a substance use disorder, plus mental health, and there’s no services available for that client.

SSC Supervisor #4

*Lack of community support* A few SSCs highlighted a unique and potentially important barrier in their rural communities. Despite the immense harm caused by opioid use in Appalachia, there was the perception that in general, communities did not acknowledge OUD as a chronic relapsing medical condition and knew little about the effectiveness of treatment (n = 5). The perception of substance use disorders in rural communities was described by one SSC, as follows:

I guess you can say maybe the community perspective of mental health and substance abuse. It’s the elephant in the county that they refuse to see…. People don’t perceive it well at all. ‘Just quit, you’re an addict.’ It’s not really welcome as much. It’s hard go to treatment or to seek treatment when you’re embarrassed to even enter a room. And even in small counties, you may see a person next to you that is your neighbor. That’s even more embarrassing in itself.

SSC #14

Not only did the community stigma serve as a barrier, but one SSC stated there was little willingness to have addiction services located in the communities- a need that was critical given the lack of treatment resources, as previously discussed.

You know if there is some type of treatment center depending on, nobody wants that next to door to their business. Nobody wants that next door to their neighborhood, whatever type of treatment it is because then you got the folks that loiter outside, and they smoke. And so then it becomes a barrier to providing resources.

SSC Supervisor #3

### System level barriers

*Lack of transportation* All but one (n = 14) of the SSCs mentioned transportation was a significant barrier to their clients accessing treatment in the community upon release, particularly the lack of public transportation. Rural clients often were forced to rely on family networks for transportation to appointments, which can be even more problematic given the distance and rough terrain to reach providers in Appalachian counties. This was often cited as a source of stress for both the client and the family, as described in the following:

Transportation is a big deal. Even if I have a client whose nearest provider is the next county, which may only be twenty minutes or something twenty miles, we don’t have mass transit so everyone can’t just hop on a bus and get a ride. And a lot of the time some of the providers are actually in some of the more rural counties. So there’s no reason for grandma to have to go to the store in that county and thus give them a ride to that area…. So if there’s not a reason for them to go to that county, then they don’t have a ride there and so even if we can go through the steps of getting them an appointment, and getting their medical insurance signed up, and getting them an appointment- then a lot of times their ride falls through.

SSC # 10

Limited transportation was primarily explained as a result of rural sprawl. SSCs also noted that many of their clients lacked a driver license and most experienced economic strain, which meant the entire household shared one car. Individuals were also limited due to the lack of public transportation in their communities:

Even if you’re highly motivated there’s not a bus to get on. Someone has to have a car and they have to be willing, and they have to be willing on the day that you need them to go.

SSC Supervisor # 3

As the quote above illustrates, the lack of public transportation was perceived as a source of strain and potentially de-motivating even among the most motivated individuals. However, SSCs also mentioned the lack of driver licenses and reliance on social networks for rides, indicating a fluidity of this barrier as both occurring on the individual level yet strained by larger system structures in Appalachia.

*Treatments are cost prohibitive* OUD treatments were often viewed as cost prohibitive for clients, in part because of larger healthcare and pharmaceutical infrastructure issues. Ten of the SSCs mentioned treatment was hard to obtain due to cost, and the majority of these referred specifically to MAT, as discussed by one SSC when asked about the lack of use of MAT by her clients:

Well I think one, is the cost. Unfortunately I don’t think many know about all of the options for the medically assisted [treatment]. It’s primarily just the common Suboxone^®^ and methadone and those are very expensive generally. They just can’t afford that. I’ve had several start in that program. And I mean, 300 to 400 dollars a month, that’s just very hard to maintain.

SSC #15

*Uncertain future of the Affordable Care Act (ACA)* While all of the SSCs mentioned insurance in some capacity, some situated their comments within a context of the Medicaid program, Kentucky’s expansion of Medicaid to cover uninsured low-income adults, and the ACA. Nine of the SSCs mentioned how changes to insurance, ease of Medicaid enrollment, or the ACA specifically improved access to care for their clients, as illustrated here:

Before we had changes with the insurance we would have trouble finding payer sources. But the change in the Affordable Care Act helped with that.

SSC Supervisor #4

At the time of the interviews, the nation was in the midst of the new presidency of Donald Trump who made a campaign promise to repeal the Affordable Care Act, and during data collection, the US Congress voted on a number of measures to change the ACA. This was reflected in some of the interviews, as SSCs perceived changes to the current system as a future barrier to access for clients:

Right now it’s really good [the health care system] because they’re eligible for health insurance but if that changes that will really effect what they’re eligible for and really will affect our job and what we can do for them.

SSC #6

## Discussion

This study of SSCs in Appalachian communities found several barriers to treatment for individuals with OUD. These barriers were identified at the levels of individuals, interpersonal networks, institutions and organizations, the community, and systems within the social ecological model. Examination of the barriers within this framework provided a nuanced method to promote suggestions for reducing barriers.

At the individual level, clinicians identified their clients as high-risk users who were often unmotivated to engage in treatment. Injection drug use practices facilitates the transmission of bloodborne infections (e.g., HIV, HCV), and this concern was noted among the SSCs. Young persons who inject drugs may be at increased risk of infections, owing to sharing needles and drug preparation equipment with sexual partners or pooling money to buy drugs and consequent needle sharing [42, 43]. One of the SSCs mentioned the youth and limited life experiences of their high-risk clients, and research suggests understanding the social context and relationships of young persons who inject drugs could assist in lowering risk among this population [42]. Extant research has indicated motivation represents a challenge to getting individuals to access care [44]. However, this research has also demonstrated that those who may be ‘least motivated’ typically have the most chronic health problems including injection drug use [44]. Thus while perceived as stigma and lack of motivation by SSCs, there could be some individuals who have become disenfranchised with the lack of services available to them or a feeling of being caught in the criminal justice system which is not primarily focused on treatment. Perceived lack of motivation could also stem from stigma and embarrassment for individuals who first started using prescription medications stemming from legitimate medical concerns before progressing to misuse and OUD.

Network level barriers included homophilious client networks with limited substance use disorder treatment knowledge among network members. The SSC-client relationship could be expanded to include conversation or even take-home educational resources for families in order to help educate client networks. A network-based intervention could promote SSCs as social influencers who facilitate treatment, recovery, and healthy lifestyles not only for their clients, but also have trickle-down positive effects to their client’s networks. However, the institutional and organizational barriers of high SSC caseload and travel burdens would need to first be overcome in order for SSCs to have the availability and resources to provide such an intervention.

Institutional and organizational barriers included high SSC caseload and lack of probation/parole officer education about OUD. Institution specific barriers to the DOC (e.g., caseload) would require additionally allocated resources to reduce burden for SSCs. Additionally, a clear and perhaps easy to accomplish improvement would be the provision of substance use education, to include MAT, for probation and parole officers. A general education seminar would take little resources or time away from officer supervision duties and could be added to current training mandates. Provision of education to overcome discrepancies in employee education is particularly important given the findings that often the DOC was one of the only service providers available in rural counties. The education of officers may assist with reversing the prohibition of MAT by some officers. At a more institutional level, the SSCs reported that judges also prohibited the use of MATs and overcoming this challenge would require the support of DOC, as well as possibly state legislation in a more comprehensive approach.

At the community level, SSCs reported that the community lacked both treatment resources and community-level support. The lack of treatment resources in rural areas is by no means a new phenomenon. In addition to the support for the use of telemedicine in rural areas, certain advances in MAT may be advantageous for individuals with OUD who would benefit from MAT as part of their treatment plan [14]. The advent of time-release formulas (e.g., Sublocade^®^, Vivitrol^®^) may reduce the frequency of transportation barriers for eligible individuals. Combined with telemedicine for psychosocial counseling, rural clients could fare better even in the face of reduced community resources.

Previous research has found health care providers have reported difficulties in building community relationships in rural areas [45]. Similar to suggestions for overcoming physician community distrust, SSCs who live and actively participate in their communities in informal ways (e.g., church, local festivals) may be presented with informal opportunities to educate the community [45]. More formal ways to educate the community could include the DOC encouraging and providing time for SSCs, or even a specified community liaison, to be present at community events in more formal capacities (e.g., educational booth, hosting an event, inter-agency collaborations with public health departments).

Related to community support was the potential for community stigma. Rural clients are more likely to have concerns related to confidentiality due to smaller networks [18, 20]. As one author stated, rural communities are like “fishbowls” in that the attendance at treatment is observed by community members, and privacy can be difficult to protect [34]. Integrating treatment such as MAT into primary care settings could assist with this barrier. Further, increased general education for the community could assist in removing the social stigmas associated with substance use disorders, similar to public health approaches of education for other chronic health conditions (e.g., diabetes, heart disease).

The ease of access to opioids also presented a challenge to clients’ treatment. This challenge has been reported nationwide, but can be particularly pronounced in areas where nonmedical prescription pain relievers offer a form of social capital [46], as spurred by the years of ‘pill mills’ proliferating rural areas. Further, nationwide trends indicate that cost, ease of access, and policy have resulted in transition from nonmedical prescription pain relievers to less expensive heroin [47, 48]. This issue of supply and access is one that still remains to be addressed via policy and law enforcement efforts.

Finally, at the systems level, the barriers to care identified by SSCs included lack of transportation, high-cost of treatments, and uncertainty of the ACA. Having a driver’s license has been shown to significantly increase the likelihood of health care utilization [17]. Lack of a license among clients may not be due to individual characteristics, but rather due to systemic issues such as cost or statutory prohibition of licensing [49]. Provision of rides from family and friends also increases the likelihood of chronic care visits; [17] however, as network barriers here indicate, social networks may not always perceive OUD as a chronic health condition in need of care and support. Access to transportation could be improved in rural areas, if current medical transportation that is often limited to older or disabled adults was expanded to include the current OUD and other substance use disorder populations. However, even these options are not without significant barriers [17] (e.g., expensive to counties, time-consuming). New advances in telemedicine could be particularly important among rural individuals with OUD [50]. Since telehealth still carries potential infrastructure and cost burdens, the use of mobile treatment providers would be additionally beneficial.

While the high cost of treatment has been noted by other research, [15, 20] a novel contribution of the current interviews was the SSCs perspective and concern over the ACA. While the individual mandate portion of the ACA was repealed in October 2017, the ACA and the portions related to Medicaid remain unchanged as of this writing. Studies continue to find the ACA has beneficial effects through uninsured rate reductions among vulnerable populations [51] and in rural areas [52, 53]. Policymakers who are dedicated to addressing the opioid epidemic should continue to support the ACA and expansion of treatment resources.

This study was not without limitations, which future research should consider. The study only assessed barriers as perceived by SSCs, a critical perspective given their role yet additional barriers may be perceived from the perspectives of clients. The perceptions of clinicians may be biased, incomplete, or out of touch with the everyday lived experiences of the clients served. It is also possible the responses were biased as a result of the SSC’s projecting desired outcomes that were not reflective of all stakeholders. This study was limited to SSCs in one geographic location—Appalachian Kentucky. While all SSCs in this area were included in the study, the sample size was limited. Future research should consider the cultural nuances of other rural locations, such as the Mississippi Delta or the rural Southwest. Treatment needs and barriers to access may differ for people released from prison to other rural regions of the U.S. In addition, there is wide-variation in the operating procedures of each state’s Department of Corrections, some of which may not have staff who serve in a role similar to Kentucky DOC’s SSC. However, Kentucky DOC has implemented several innovations to address the treatment needs of people who are under criminal justice system supervision. These strategies include establishing SSC positions to promote linkages to behavioral health services at re-entry, offering extended release naltrexone in prison and jail at re-entry for clinically eligible clients who completed a correctional substance use disorder treatment program, and establishing Recovery Kentucky which created 13 community-based centers to provide housing and recovery services for 2000 Kentuckians simultaneously. It is important to keep in mind the varying educational foundations that were found to exist within this system, and future research could consider how perspectives and services vary by SUD training and education. In addition to identification of barriers from the perspective of others (e.g., clients, probation and parole officers), future research should consider interventions that seek to overcome the barriers identified. For example, the use of telemedicine services as well as mobile treatment providers could be particularly advantageous in rural communities. Network and community-level interventions focused on education of OUD and MAT would additionally be beneficial.

## Conclusions

The current study offered the perspective of clinicians in assessing the barriers to treatment for opioid use in resource-limited Appalachian Kentucky. Through utilization of the social ecological framework, these barriers were situated as individual, interpersonal, institutional/organizational, community, and system-level. The SSCs in Appalachian Kentucky cited barriers to treatment to include:, high risk use, stigma and lack of motivation, homophilious networks, networks with limited knowledge of treatment, high caseloads, lack of substance use disorder education for parole/probation officers, limited availability of treatment resources, ease of access to opioids, lack of community support, lack of transportation, cost prohibitive treatments, and an uncertain future of the Affordable Care Act. Identification of these barriers from the perspective of DOC employed SSCs was critical, as these clinicians are directly responsible for post-release referrals and after-care services. Identified suggestions provide real-world opportunities for improving access to treatment in Appalachia.

## Abbreviations

OUD: opioid use disorder
SSC: social service clinician
DOC: Department of Corrections
MAT: medications for addiction treatment
ACA: Affordable Care Act
